# *QuickStats:* Repeat* Birth Rates for Teens,^†^ by Urbanization Level of County^§^ — National Vital Statistics System, 2007–2016

**DOI:** 10.15585/mmwr.mm6735a7

**Published:** 2018-09-07

**Figure:**
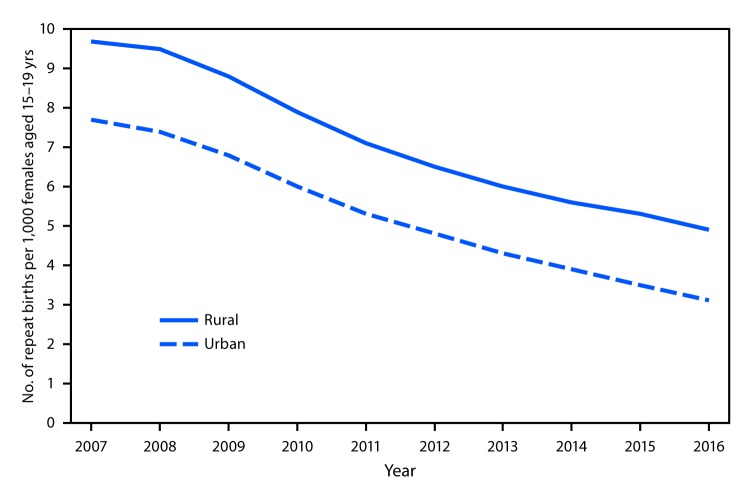
From 2007 to 2016, the rate for repeat births for females aged 15–19 years significantly declined in both rural and urban counties. Repeat birth rates declined 49% in rural counties (from 9.7 in 2007 to 4.9 in 2016) and 60% in urban counties (from 7.7 in 2007 to 3.1 in 2016). However, the rate in rural counties was significantly higher than the rate in urban counties for each year from 2007 through 2016.

**Source:** National Vital Statistics System, Natality, 2007–2016. https://www.cdc.gov/nchs/nvss/births.htm.

